# Gut mycobiome dysbiosis contributes to the development of hypertension and its response to immunoglobulin light chains

**DOI:** 10.3389/fimmu.2022.1089295

**Published:** 2022-12-29

**Authors:** Yeqing Zou, Anxing Ge, Brako Lydia, Chen Huang, Qianying Wang, Yanbo Yu

**Affiliations:** ^1^ School of Basic Medicine, Jiangsu Vocational College of Medicine, Yancheng, Jiangsu, China; ^2^ Administration office of science and technology, Jiangsu Vocational College of Medicine, Yancheng, Jiangsu, China; ^3^ Community center, Kumasi, Ashanti Region, Ghana; ^4^ Department of Geriatrics, Affiliated Xinchang Hospital, Wenzhou Medical University, Wenzhou, Zhejiang, China

**Keywords:** gut mycobiome, prehypertension, hypertension, immunoglobulin light chains, kappa, kappa (κ) FLC, lambda (λ) FLC

## Abstract

**Objectives:**

Human gut microbiome has gained great attention for its proposed roles in the development of hypertension. The fungal microbiome in the human gut (i.e. the mycobiome) is beginning to gain recognition as a fundamental part of our microbiome. However, the existing knowledge of human mycobiome has never revealed the association between gut mycobiome and hypertension. It is known that inflammation and immunity contribute to human hypertension. Here, we sought to investigate whether gut mycobiome could predict the development of hypertension and its association with immunoglobulin light chains.

**Methods and materials:**

Participants were classified into three cohorts: prehypertension (pre-HTN), hypertension (HTN), and normal-tension (NT) based on their blood pressure. Fresh samples were collected, and the ITS transcribed spacer ribosomal RNA gene sequence was performed. An immunoturbidimetric test was used to examine the serum levels of immunological light chains.

**Results:**

Subjects in both of the states of pre-HTN and HTN had different fungal microbiome community compared to the NT group (FDR<0.05). Slightly higher levels of fungal richness and diversity were observed in the groups of pre-HTN and HTN. The relative abundance of *Malassezia* increased in the HTN group compared to that in the NT group, and the relative abundance of *Mortierella* enriched in the NT group. For the pre-HTN group, the relative abundance of *Malassezia* was positively associated with serum the concentration of light chain (LC) κ (r=0.510, P=0.044); for the HTN group, the relative abundance of *Mortierella* was positively associated with the serum concentration of LC κ (P<0.05), the relative abundance of *Malassezia* was positively associated with both the serum concentrations of LC κ and LC λ (r>0.30, P<0.05).

**Conclusions:**

Our present study demonstrated that gut fungal dysbiosis occurred in the state of prehypertension, and fungal dysbiosis can predict the dysregulation of serum light chains in hypertension patients. Further study on modulating gut fungal community should be focused on balancing the immunological features in hypertension.

## Introduction

Estimates suggest that 31.1% of adults worldwide had hypertension (1). Hypertension is the leading cause of cardiovascular disease and premature death worldwide (1). The pathogenesis of hypertension is known to involve a diverse range of contributing factors including environmental and inflammatory forces (2). There is mounting evidence that humans and models of animals support the role of the gut microbiome, a key interface between the body and the environment (3), in the development of hypertension (4–6). For example, the findings from Sun Set al. supported that there were associations between hypertensive patients with gut microbial community diversity and taxonomic composition (5). Li J et al. demonstrated that patients with primary hypertension and pre-hypertension had a decreased microbial diversity and different gut enterotypes compared to healthy controls (6).

Most studies describing the human gut microbiome in health states have been focused on the bacterial component, but the fungal microbiome (i.e., the mycobiome) is beginning to gain recognition as a fundamental part of our microbiome (7). Dysbiosis, the alterations in diversity, abundance, and functionality in the gut mycobiome has been implicated in several diseases. Jayasudha R and his colleague found that dysbiosis in the gut mycobiome in people with type 2 diabetic mellitus (T2DM) or diabetic retinopathy compared to healthy subjects (8); Demir M et al. demonstrated that non-obese patients with non-alcoholic-fatty-liver-disease and more advanced disease have a different fecal mycobiome composition to those with mild disease (9). Like the gut microbiome, gut mycobiome is affected by food habit of fecal sample donors, such as plant-or animal-based, phytoestrogens enriched plant products and fat-rich diets affect the colonization of certain fungal species in the mammalian gut (10).

Experimental studies implicate inflammation and immunity contribute to human hypertension (11). The inflammatory pathway is responsive to angiotensin receptor ligation and culminates in the translocation of nuclear factor-κ light chain (LC) enhancer of activated B cells to the nucleus, and the activation of B cells by angiotensin II potentiates target-organ damage in hypertension (12).

It is demonstrated that the activation of the immune system either directly or indirectly impacts the gut microbiome (13). However, there was no evidence in the current study that the gut mycobiome dysregulated in hypertension.

It is known that T2DM and fatty liver disease are related to impaired metabolic homeostasis and can be regarded as a metabolic disorder (14, 15). Like T2DM and fatty liver disease, hypertension is also associated with metabolism disorders (16). As patients with T2DM and fatty liver disease have a distinct mycobiome in the gut, we questioned whether the mycobiome profile in patients with hypertension is different from healthy population. To contribute the exiting knowledge of the human mycobiome, we investigated the gut mycobiome of populations with pre-hypertension and hypertension.

## Methods and materials

### Study cohort and participants recruitment

Participants were classified into three groups according to their blood pressure (BP), namely, pre-HTN (n=38), HTN (n=46), and NT (n=34). The presence of HTN was confirmed as a systolic blood pressure (SBP) ≥140 mmHg or a diastolic blood pressure (DBP) ≥90 mmHg and/or use of antihypertensive medication; pre-HTN was defined as an SBP of 120–139 mmHg or a DBP of 80–89 mmHg without the use of antihypertensive medication; Normal-tension BP (NT) was defined as an SBP ≤ 120 mmHg or a DBP ≤80 mmHg (17). According to patients baseline SBP and DBP, the patients in the HTN group were categorized into different phenotypes. To be specific, isolated systolic hypertension (ISH; SBP≥140 and DBP<90mmHg), isolated diastolic hypertension (IDH; SBP<140 and DBP≥90mmHg), systolic-diastolic hypertension (SDH; SBP>140 and DBP≥90mmHg) (18). BP was measured in a sitting position by nurses. Three readings were recorded at 5-min intervals with a random-zero mercury column sphygmomanometer, and the average was taken as the final measurement (6). Exclusion criteria for participants were as follows: acute intercurrent disease and infections, cancer, stroke, peripheral artery disease, heart failure, renal failure, kidney damage, diabetes, pregnancy, breastfeeding, elevated body temperature or white blood cell count, and those who used antibiotics, probiotic or immunosuppressive drugs within 60 days before enrollment were excluded from the present study. The ethics committee of the Affiliated Xinchang Hospital approved this study. Informed consent was provided by all subjects before their inclusion in the study.

### Sample collection and procession

The participants were instructed to collect their fecal samples to a sterile container and pick approximately 30mg of feces to a sterile bottle which contained 1000μL lysis buffer composed of Tris 0.1mol/L (pH 8.0), 2 mM EDTA, and 2%SDS (Guhe Health. com., Hangzhou, China), and stored at −80°C until further processing.

The blood samples for detecting immunological LC were collected on the day of fecal samples collection. An immunoturbidimetric test was used to assess serum levels of immunological features on the day of sample collection (AU5400; Beckman Coulter, USA). Information on clinical manifestations, concurrent diseases, and blood parameters were assessed by reviewing clinical records, medical interviews and face to face interview. In addition, the nutrient intake was assessed using a Chinese Food Frequency Questionnaire (19).

### Fungal DNA extraction

DNeasy PowerSoil Pro Kit was used to isolate fungal genomic DNA from fecal samples according to the manufacturer’s instructions in a biological safety cabinet (QIAGEN, Germany), with additional glass-bead beating steps performed using a Mini-Beadbeater (FastPrep; Thermo Electron, Boston, MA, United States). The amount of DNA was determined using a NanoDrop ND-1000 spectrophotometer (Thermo Electron). The integrity and size of DNA were verified by electrophoresis on a 1.0% agarose gel containing 0.5 mg/ml ethidium bromide. All DNA samples were stored at −20°C prior to further analysis.

The ITS regions were amplified using ITS1F (5’-CTTGGTCATTTAGAGGAAGTAA-3’) and ITS2 (2043R; 5’-GCTGCGTTCTTCATCGATGC-3’) primers (20). All PCR reactions were performed using Phusion High-Fidelity PCR Master Mix (Thermo Scientific Inc., Waltham, MA, USA) according to the manufacturer’s protocol and approximately 50 ng extracted DNA per reaction. Thermocycling conditions were set at 98*°C* for 15 sec for 1 cycle, then 98°C for 15 sec, 58°C for 15 sec, then 72°C for 15 sec for 30 cycles, followed by a final extension at 72°C for 60 sec for 1 min. Negative DNA extraction samples (lysis buffer and kit reagents only) were amplified and sequenced as contamination controls. Amplified products were purified using Agencourt AMPure XP beads (1 volume; Beckman Coulter, Pasadena, CA) and samples were run on a 1% agarose gel in order to size-select gel slices around 430 bp. The amount of DNA was determined using a Qubit 2.0 Fluorometer (Life Technologies, Carlsbad, California, US). Sequencing was performed with 2×150bp on a Novaseq 6000 platform ((Illumina Inc., San Diego, CA, USA).

### Bioinformatic analysis

The ITS sequence dataset was merged and demultiplexed into per-sample data using QIIME (V1.9.1) with default parameters (21). Raw sequencing reads with exact matches to the barcodes were assigned to respective samples and identified as valid sequences. The low-quality sequences were filtered through the following criteria: sequences of specified length of <150bp, sequences of an average Phred scores of <20, sequences of ambiguous bases, and sequences of mononucleotide repeats of >8 bp. Paired-end reads were assembled using Vsearch (V2.4.4; -fastq_mergepairs -fastq_minovlen 0). Operational taxonomic unit (OTU) picking included Dereplication (-derep_full length), cluster (-cluster_fast, -id 0.97), detection of chimeras (-uchime_ref). A representative sequence was selected from each OTU using default parameters. OTU taxonomic classification was conducted by Vsearch searching the representative sequences set against the UNITE 12_11 (https://unite.ut.ee) database (22).

An OTU table was further generated to record the abundance of each OTU in each sample and the taxonomy of the OTUs. A minimum library size was chosen to rarefy the OTUs in our present study, as it is critical to normalize the OTU table to eliminate any bias due to differences in the sampling sequencing depth. And total sum scaling (TSS) was applied to transform the OTU table into relative abundance by dividing the number of total reads of each sample. OTUs containing less than 0.01% of total sequences across all samples were discarded.

Sequence data analysis was performed using QIIME and R package (V3.2.0). OTU-level alpha richness and diversity indices, including Chao 1, Shannon and Simpson, were calculated using the OTU table. Beta diversity analysis was performed to investigate the structural variation of fungal communities across samples using Bray-Curtis metrics and visualized *via* principal coordinate analysis (PCoA) based on permutational multivariate analysis of variance [PERMANOVA] calculated by ‘adonis’ function.

### Statistical analysis

Pearson’s Chi-square or Fisher’s exact tests were used with categorical variables; Student’s *t* test and ANOVA were used on normalized continuous variables and Wilcoxon rank-sum test on non-normal continuous variables. The *P*-value was adjusted for multiple comparisons using the Benjamini–Hochberg (BH) false discovery rate (FDR). Pearson correlation analysis was performed on the abundant bacterial genera (>1% relative abundances) and LC κ, LC λ and κ/λ ratio that differed between groups.

## Results

We collected fecal samples from 38 subjects with pre-HTN, 46 with HTN and 34 gender-age-BMI matched NT (**Table 1**). Compared to the NT group, both of the groups of pre-HTN and HTN had higher SBP, as well as higher DBP (P<0.05; **Table 1**). In addition, the groups of Pre-HTN and HTN had higher rates of type 2 diabetic mellitus (T2DM) and coronary heart disease than those in the NT group (P<0.05). When the LCs were assessed, the groups of pre-HTN and HTN had higher levels of κ/λ ratio, as well as LC κ and LC λ compared to the NT group (P<0.05; **Table 1**). When the nutrient intake was compared among the groups of pre-HTN, HTN and NT, we did not observe significant difference (P>0.05; **Table S1**).

**Table 1:** Demographic and clinical characteristics of the study population.

Valuables	Value for corhort (n^a^)^b^ or statistic			
	pre-HTN (n=38)	HTN (n=46)	NT (n=34)	*p* value^c^
Gender				
Male [no. (%)]	19 (50.00)	23 (50.00)	17 (50.00)	1.000
Female [no. (%)]	19 (50.00)	23 (50.00)	17 (50.00)	1.000
Married status [no. (%)]	36 (94.74)	43 (93.48)	30 (88.24)	0.566
Age	56.68 ± 16.60	57.24 ± 17.40	62.18 ± 16.67	0.328
Body mass index (kg/m^2^)	27.25 ± 7.29	74.63 ± 36.14	24.26 ± 3.41	0.492
History of drinking	2 (5.26)	4 (8.70)	2 (5.88)	0.802
History of smoking	1 (2.63)	2 (4.35)	0 (0.00)	0.324
Vegetarian	1 (2.63)	2 (4.35)	0 (0.00)	0.474
Duration of HTN (years)				/
Less than 1 year [no. (%)]	/	5 (10.87)	/	
1-3 years [no. (%)]	/	12 (26.09)	/	
3-5 years [no. (%)]	/	11 (23.91)	/	
More than 5 years [no. (%)]	/	10 (21.74)	/	
Systolic blood pressure (mmHg)	120.92 ± 7.98^d^	157.09 ± 19.25^e^	87.27 ± 22.14	<0.05
Diastolic blood pressure (mmHg)	86.58 ± 8.14^d^	96.2 ± 10.99^e^	70.73 ± 10.09	<0.05
Estimated glomerular filtration rate (mL/min/1.73m^2^)	92.06 ± 35.48^d^	90.01 ± 25.26^e^	116.23 ± 56.41	0.510
Currently taking anti-hypertension agent	/	30 (65.21)	/	/
Comorbidities				
Type 2 diabetic mellitus [no. (%)]	8 (21.05)^d^	15 (32.61)^e^	0	<0.05
Coronary heart disease [no. (%)]	5 (13.16)^d^	7 (15.22)^e^	0	<0.05
0κ/λ ratio	1.82 ± 0.31^d^	1.94 ± 0.48^e^	1.53 ± 0.34	<0.05
LCκ	8.29 ± 6.05^d^	7.43 ± 3.03^e^	2.98 ± 0.46	<0.05
0LCλ	4.37 ± 2.73^d^	3.95 ± 1.62^e^	1.67 ± 0.42	<0.05

a n, no. of subjects.

b Mean ± SD or no. (%).

c Fisher’s exact test was used for categorical variables and one-way analysis of variance was used to compare continuous variables.

d Statistically significant difference was found between groups of Pre-HTN and NT.

e Statistically significant difference was found between groups of HTN and NT.

HTN, hypertension; NT, normotension; pre-HTN, pre-hypertension.

**Figure 1:**
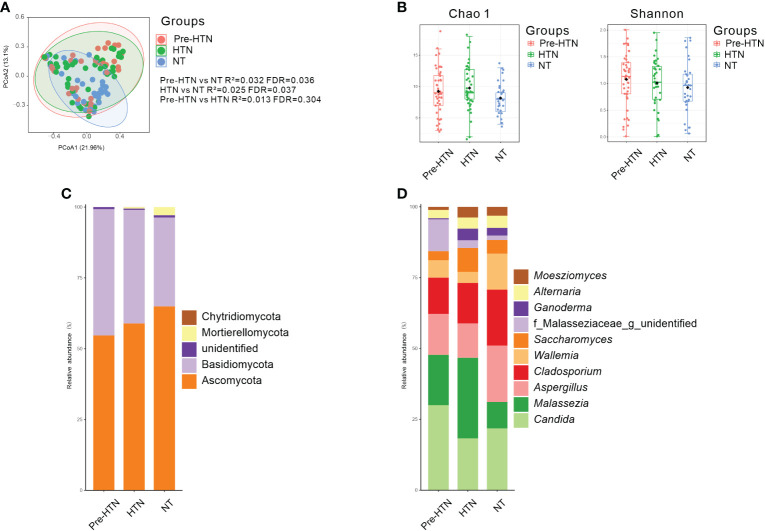
Bacterial composition, Venn, bacterial diversity, and phylum and genus composition in the urine from subjects of pre-HTN, HTN and NT. **(A)** PCoA based on Bray-Curtis distances ASV level was performed to compare microbial community among groups of pre-HTN, HTN and NT. The 95% confidence ellipse is drawn for each group. Permutational multivariate analysis of variance (PERMANOVA) was performed for statistical comparisons of samples in the two groups. *P* value was adjusted by the Benjamini and Hochberg false discovery rate (FDR). **(B)** Bacterial richness and diversity measured by Chao 1 and Shannon index were calculated at ASV level. Wilcoxon rank-sum test was performed and adjusted by Benjamini and Hochberg false discovery rate (FDR). **(C, D)** Fungal profile at the phylum and genus level. Only the top 10 most abundant genus are shown. HTN, hypertension; NT, normotension; pre-HTN, pre-hypertension.

When comparing to the NT group, we found that the fecal mycobiome of both pre-HTN and HNT groups differed (PERMANOVA, R^2 =^ 0.032, FDR=0.036 and R^2 =^ 0.025, FDR<0.037, respectively; **Figure 1A**). Anti-hypertension agent was not a confounding variable in explaining the difference between HNT and NT groups, as fecal mycobiome of the medication users and non-users did not differ (PERMANOVA, R^2 =^ 0.023, FDR=0.414; **Figure S1**). The mycobiome of pre-HTN group did not differ from that in HTN (PERMANOVA, R²=0.013 FDR=0.304; **Figure 1A**). The fecal mycobiomes of pre-HTN and HTN subjects were slightly richer and more diverse than those of their respective NT subjects (*Wilcoxon rank-sum test*, FDR>0.05; **Figure 1B**). When we categorized the HNT patients based on their blood pressure phenotypes, no differences were found among the sub-phenotypes of ISH, IDH and SDH (PERMANOVA, R²=0.059 FDR=0.051; **Figure S2**).

When the bacterial phyla were assessed **Figure 1C**, Ascomycota dominated in groups of pre-HTN, HTN and NT, it was accounted for 54.68%, 58.90%, and 64.94%, respectively. Basidiomycota was also dominated in groups of pre-HTN, HTN and NT, it was accounted for 44.58%, 40.12%, and 31.38%, respectively. At the bacterial genus level **Figure 1D**, the pre-HTN group was dominated by *Candida* (29.97%), *Malassezia* (17.78%), *Aspergillus* (14.46%), *Cladosporium* (12.79%), and a member of the bacterial family of Malasseziaceae (11.20%). The dominated bacteria genus in the HTN group were somewhat different from those in the pre-HTN group, such as *Malassezia* was accounted for 28.45%, followed by *Candida* (18.26%), *Cladosporium* (14.30%), *Aspergillus* (12.13%), and *Saccharomyces* (8.46%). The NT group was dominated by *Candida* (21.80%), *Aspergillus* (19.88%), *Cladosporium* (19.77%), *Wallemia* (12.71%), and *Malassezia* (9.31%).

The classified species from the dominated fungal genera in the pre-HTN, HTN and NT groups were examined (**Table S2**), we observed that both of two species *Parapsilosis* and *Tropicalis* from *Candia* were predominated in the samples of NT group (accounted 5.90% and 2.26%, respectively).

**Figure 2:**
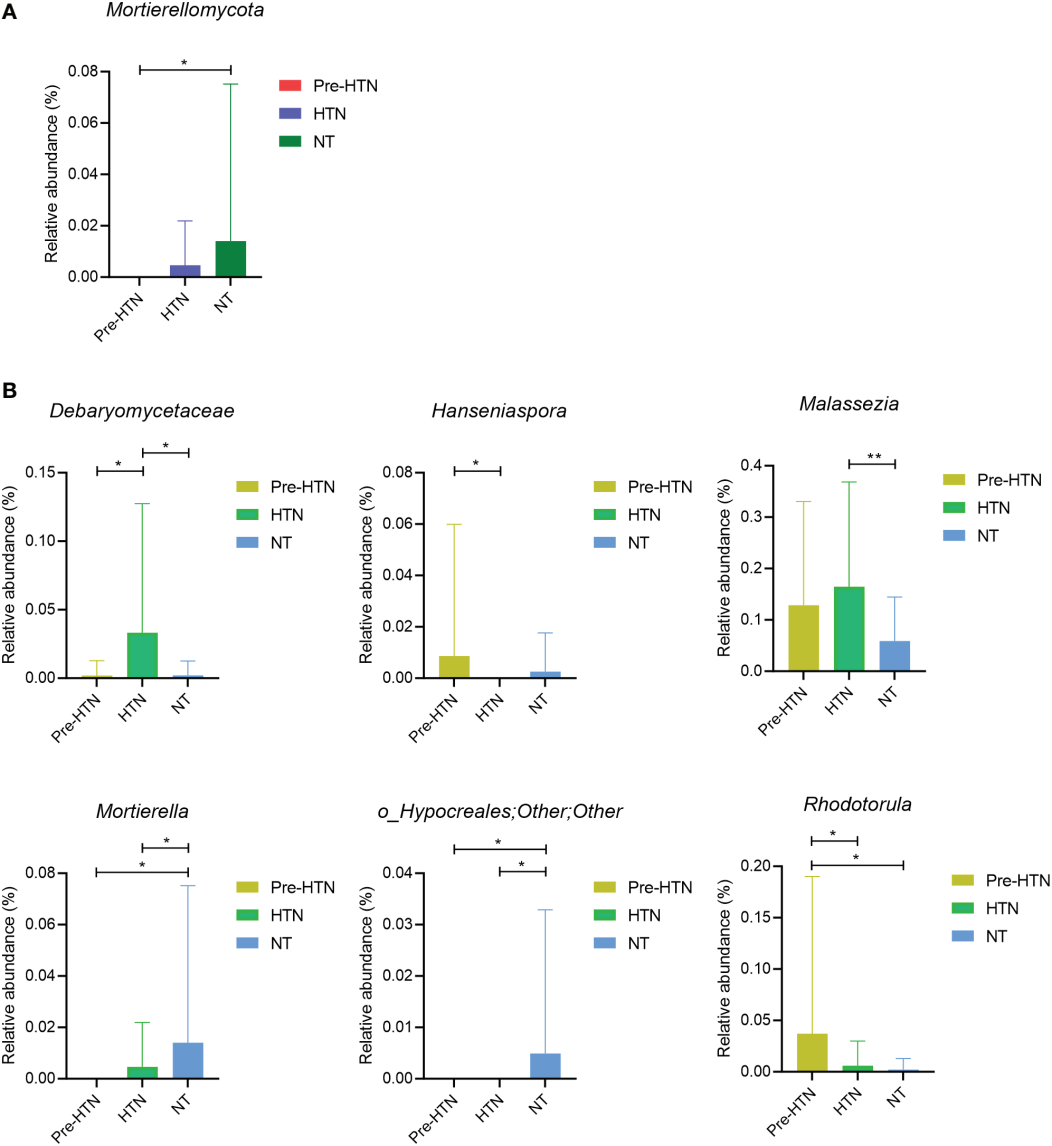
Fungal phyum and genus that were differentially abundant among groups of pre-HTN, HTN and NT. **(A)** Bacterial phylum significantly different among groups of pre-HTN, HTN and NT. **(B)** Bacterial genera significantly significantly different among groups of pre-HTN, HTN and NT. P value was calculated using Wilcoxon rank-sum test and adjusted by Benjamini and Hochberg FDR. *FDR < 0.05; and **FDR<0.01.

Next, we compared the relative abundances of bacterial phylum and the bacterial genus with above 1% of the total abundance among the three groups of pre-HTN, HTN and NT. We found that *Mortierellomycota* significantly increased in the NT group compared to the pre-HTN group (*Wilcoxon rank-sum test*, FDR<0.05; **Figure 2A**). **Figure 2B** displayed several fungal genera showing significant difference among the three groups (*Wilcoxon rank-sum test*, FDR<0.05). For example, the HTN group had higher abundance of a member of the family of *Debaryomycetaceae* than that in the groups of pre-HTN and NT. *Hanseniaspora* increased in pre-HTN group compared to HTN group. Although *Malassezia* increased with BP, the difference was only observed between HTN and NT groups. Among the three groups, the NT group had the highest abundances of *Mortierella* and a member of the bacteria order of Hypocreales.

It is known that immune cells play a role in hypertension and a previous study evidenced that there was a relationship between white blood cells and blood pressure in hypertension population (23–25). Thus, the three fungal genera differed in HTN group compared to NT group were selected to perform Pearson correlation analysis with the white blood cell types to see the gut mycobiome associations immune cells. However, no significant associations were found (P>0.05; **Table S3**).

BMI, age and eGFR are factors influencing blood pressure in hypertension patients (26–28), thus we assessed their associations with gut mycobiome in HTN patients. Pearson correlation analysis was performed between the differed fungal genera in HTN group and patient’s BMI, age, eGFR, hypertension duration, and we did not notice any significant associations (P>0.05; **Table S4**).

**Figure 3:**
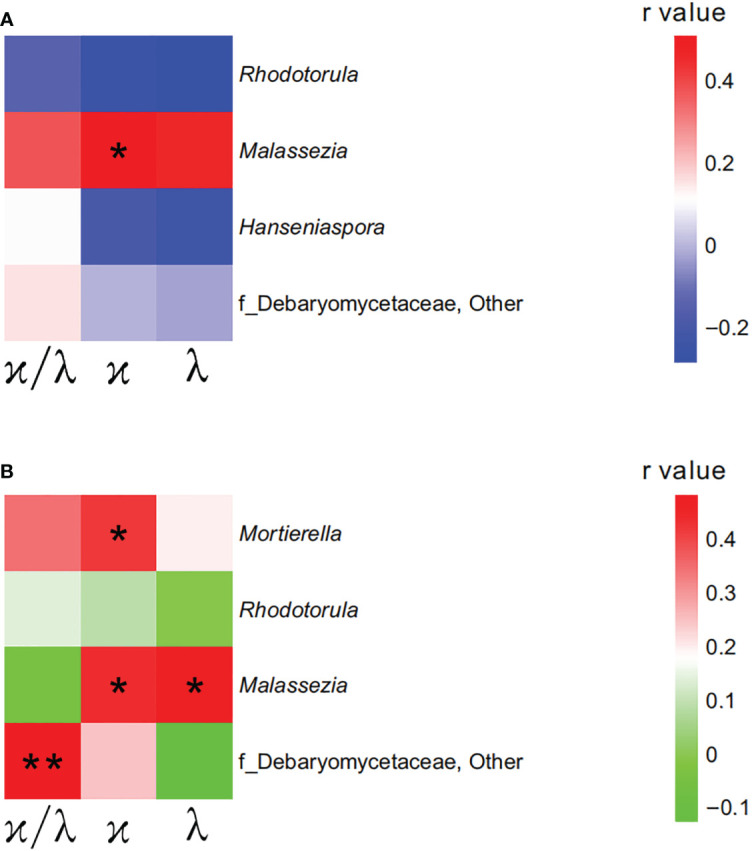
Mycobiome was associated with participants’ immunoglobulin light chains. **(A)** The heatmap depicted the association between the bacterial genera and immunoglobulin light chains showing differed in pre-HTN compared to NT. **(B)** The heatmap depicted the association between the bacterial genera and immunoglobulin light chains showing differed in HTN compared to NT. Pearson correlation analysis was performed. The correlation of two variables with values of |*r*|>0.3 and *P < *0.05 are displayed. *P < 0.05; and **P < 0.01.

As serum immunoglobulin light chains, including serum concentrations of LC κ, LC λ and their ratio of κ/λ sharply increased in the groups of pre-HTN and HTN than those in the NT group, we used Pearson correlation analysis to determine whether any of the significantly different taxa were associated with any immunoglobulin light chains and their ratio. For both pre-HTN and HTN groups, multiple associations were observed. For example, the relative abundance of *Malassezia* was positively associated with serum concentration of LC κ in the pre-HTN group (r=0.510, P=0.044; **Figure 3A**). More associations were observed in the HTN group than those in the pre-HTN group, such as the relative abundance of *Mortierella* was positively associated with serum concentration of LC κ (r=0.420, P=0.026; **Figure 3B**); the relative abundance of *Malassezia* was positively associated with both serum concentration of LC κ and LC λ (r=0.439, P=0.019 and r=0.473, P=0.011 respectively; **Figure 3B**); the relative abundance of a member of bacterial family of Debaryomycetaceae was positively associated with the ratio of κ/λ (r=0.483, P=0.009; **Figure 3B**).

## Discussion

Like the gut microbiome, our present study firstly identified that gut mycobiome composition and fungal profiles are associated with hypertension in human, as well as the gut mycobiome in patients with hypertension is shown to be an important determinant of the disordered serum light chains.

As a previous researcher claimed that there was an association of bacterial dysbiosis in the gut in patients with pre-HTN or HTN (6), we also noticed that fungal dysbiosis was lined to the participants with pre-HTN or HTN. However, no difference was found in the fungal community between the groups of pre-HTN and HTN. In their study, Li J et al. found that bacterial composition in human pre-HTN subjects was very similar to that of the HTN patients (6). These findings of the bacterial community in Li J study and the fungal community in our present study suggest that changes of both gut microbiome and gut mycobiome precede the onset of HTN.

Loss of gut bacterial diversity is associated with unhealthy states (29), including individuals with hypertension (6, 30). Inconsistent with the bacterial diversity in the gut in hypertension (6, 30), our present study demonstrated that the fungal richness and diversity tended to increase in patients with pre-HTN or HTN compared to controls, which has been evidenced by a recent study on patients with chronic kidney disease (CKD) (31). In another study, Liguori G et al. found that less bacterial operational taxonomic units (OTUs) in Crohn’s disease patients compared to healthy subjects, whereas larger fungal load was found in Crohn’s disease patients (32). As both of studies of Liguori G and ours were with small sample size, which might reduce the power of the studies and render the study meaningless, the inconsistent alterations of fungal diversity and bacterial diversity should be re-confirmed using studies with large sample size population from multiple locations.

Recently, Hu et al. reported that the dysbiosis of gut mycobiome accompanying by an increased bacterial diversity in patients with CKD (31). In their study, 65.22% CKD patients were diagnosed with hypertension (31). However, although the participants in the groups of pre-HTN and HTN in our present study with normal kidney function, the gut dysbiosis of mycobiome occurred in the states of pre-HTN and HTN. It is known that hypertension is a leading cause of CKD (20), and the occurrence of gut mycobiome in the states of pre-HTN and HTN suggests that modulation of gut mycobiome in the states of raised blood pressure may be effective in relieving the kidney damage.

Our findings suggest that *Malassezia* is a potential biomarker relating to the development of hypertension, as it was not only enriched in the states of pre-HTN and HTN, but also positively responsible for the increase of LC κ in both subjects with pre-HTN and HTN, and positively responsible for the increase of LC λ in HTN patients. It is reported that increased levels of LCs have been detected in various inflammatory disease (21, 22, 33). Recent studies have shown that LCs can bind to mast cells and, using their ability to bind antigen, facilitate activation of these mast cells and dorsal root ganglia and neutrophils. These activations can result in the release of various pro-inflammatory mediators which are believed to contribute to the development of the inflammatory disease (34–36), and inflammation plays a significant role in the pathogenesis of hypertension (11). *Malassezia* species manifest multiple proinflammatory biological properties (37), and can promote the development of inflammatory associated diseases, such as Crohn disease (38), and pancreatic cancer (39), inflammatory bowel disease (40), and skin disease (41). Thus, *Malassezia* is considered an important emerging pathogen. Our observations of the enrichment of *Malassezia* in the states of Pre-HTN and HTN and its positive connection to LCs remind us that *Malassezia* is a potential pathological fungus in hypertension. Further study involving animal models and fecal microbiome transfer from the donors of hypertension is needed to define the function of *Malassezia.*

We noticed that *Mortierella* depleted in the states of Pre-HNT and HTN compared to healthy controls. In their study, Jayasudha R and his group found that *Mortierella* was not observed in patients with T2DM or diabetic retinopathy, whereas it was present in the healthy subjects (8). Similarly, Wu N and his colleagues found that healthy subjects had an increased *Mortierella* than that in patients with gestational diabetes mellitus (42), and they also found that *Mortierella* represented a modulating effect on blood glucose level (42). From the findings in the abovementioned previous studies and our present study, we conclude that *Mortierella* might play a probiotic role in hypertension. However, it is hard to understand its positive association with LC λ in the group of HTN. Further study is necessary to investigate whether this is a protective immunological response in hypertension.

Our study has limitations. Firstly, pre-HTN and HTN patients and controls were not individually matched, there may exist additional confounders. Secondly, a single institution enrolled our local participants in our study, and it is not comparable to a multiple-center study which can appropriately powered the fungal profiles. Thirdly, although no differences were found in the nutrient intake among the three groups of pre-HTN, HTN and NT, other factors, such as physical activity (43), should be investigated and assessed their confounding effects on the gut mycobiome.

In summary, our present study firstly demonstrates the gut mycobiome profiles among patients with pre-HTN and HTN. We show that the dysbiosis of gut mycobiome occurred in the state of prehypertension, which suggest that the modulation therapy towards gut mycobiome should not only be targeted at hypertension patients, but also at the subjects in the state of prehypertension, which might prevent further elevations in their blood pressure. In addition, we noticed that the disordered immunological profiles were associated with the dysbiosis of gut mycobiome when subjects with increasing blood pressure. Microbial sensing, metabolic signalling and immune response pathways ensure the survival of gut microbiome in a microbially dominated world, and the interaction between gut microbiome and host immunity homeostasis provides essential health benefits to the host (44). Thus, further study should target the differing mycobiome community to modulate host immunity homeostasis.

## Data availability statement

The datasets presented in this study can be found in online repositories. The names of the repository/repositories and accession number(s) can be found in the article/**Supplementary Material**.

## Ethics statement

The studies involving human participants were reviewed and approved by The Affiliated Xinchang Hospital approved this study. The patients/participants provided their written informed consent to participate in this study. Written informed consent was obtained from the individual(s) for the publication of any potentially identifiable images or data included in this article.

## Author contributions

Conceptualization: YZ, YY. methodology: AG, BL, CH, QW, YY. software: CH, QW. validation: YY. writing: YY. supervision: YY. funding acquisition: YY. project administration: CH, QW, YY. All authors contributed to the article and approved the submitted version.

## Supplementary material

Supplementary Figure 1 Medication usages on fecal mycobiome. Comparison of fecal mycobiome between antihypertensive agent users and non-users. Permutational multivariate analysis of variance (PERMANOVA) was performed for statistical comparisons of samples in the two groups. *P* value was adjusted by the Benjamini and Hochberg false discovery rate (FDR).

Supplementary Figure 2 Hypertension phenotypes on fecal mycobiome. Comparison of fecal mycobiome among hypertension phenotypes. Permutational multivariate analysis of variance (PERMANOVA) was performed for statistical comparisons of samples in the two groups. *P* value was adjusted by the Benjamini and Hochberg false discovery rate (FDR). **A**bbreviations: IDH: isolated diastolic hypertension; ISH, isolated systolic hypertension; SDH: systolic-diastolic hypertension.
