# Supplementary figures and images for: Gut mycobiome dysbiosis contributes to the development of hypertension and its response to immunoglobulin light chains
Source: Front Immunol. 2022 Dec 29;13:1089295. doi: 10.3389/fimmu.2022.1089295 (PMC9835811; doi:10.3389/fimmu.2022.1089295)

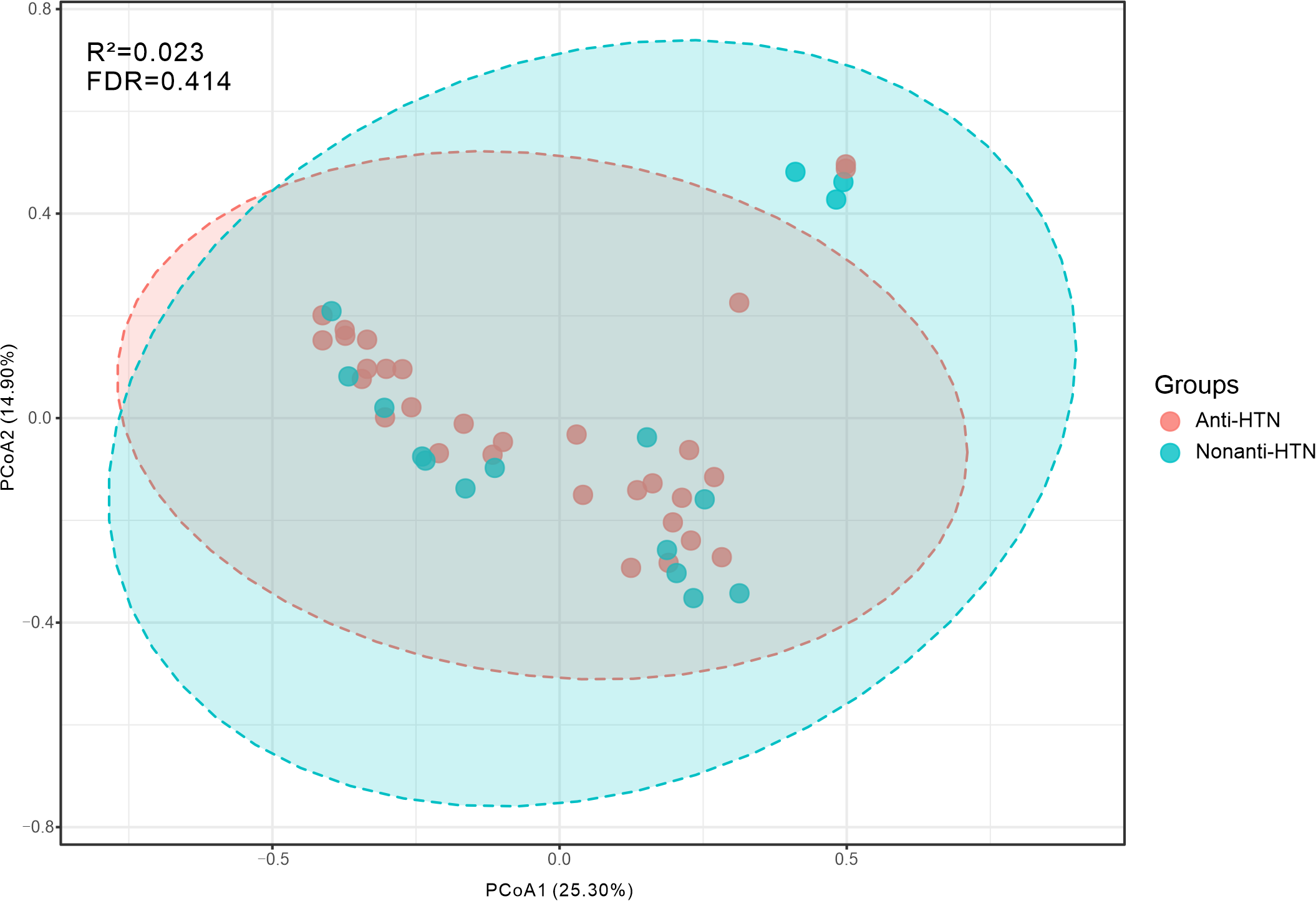

Supplement: Supplementary Figure 1 — Medication usages on fecal mycobiome. Comparison of fecal mycobiome between antihypertensive agent users and non-users. Permutational multivariate analysis of variance (PERMANOVA) was performed for statistical comparisons of samples in the two groups. P value was adjusted by the Benjamini and Hochberg false discovery rate (FDR). [file Image_1.tif]

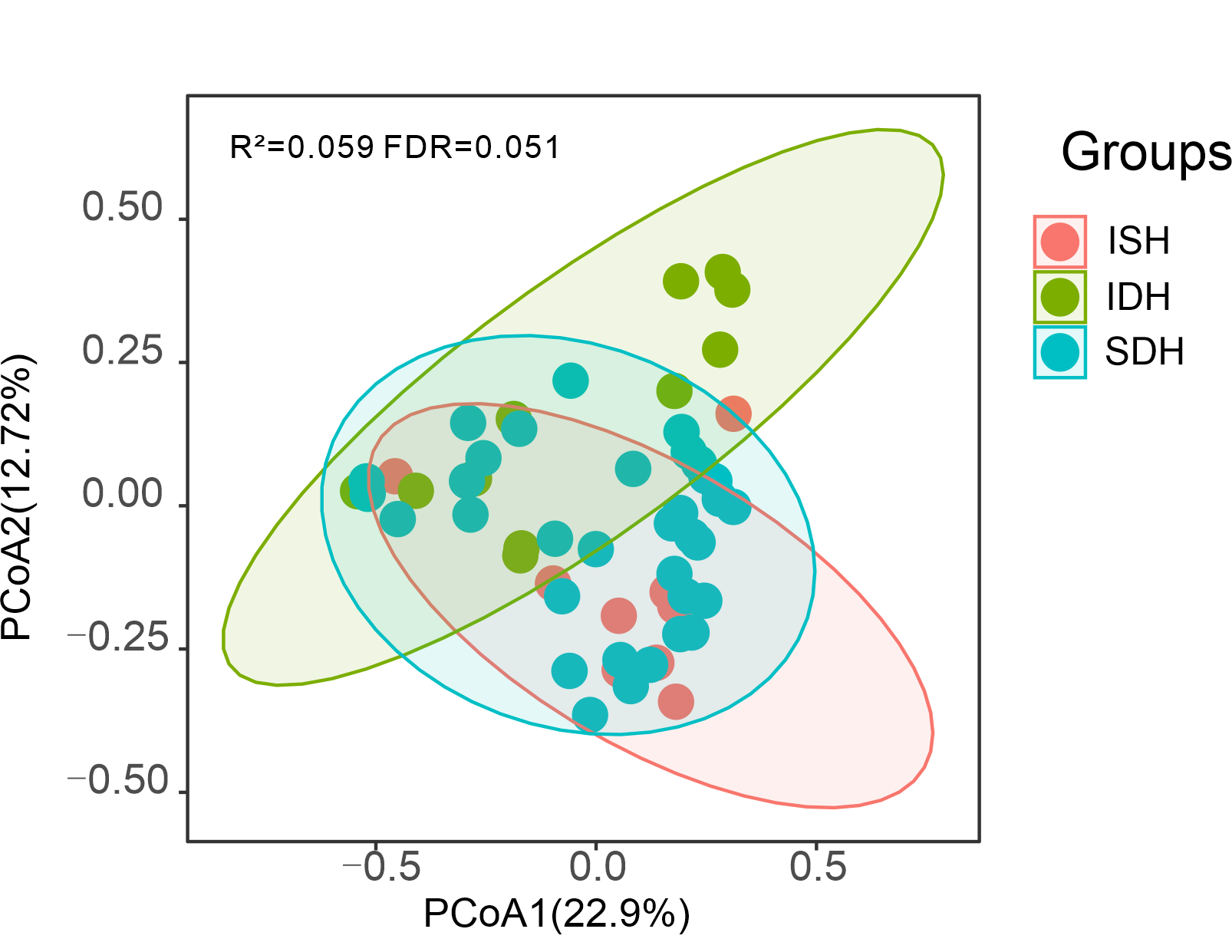

Supplement: Supplementary Figure 2 — Hypertension phenotypes on fecal mycobiome. Comparison of fecal mycobiome among hypertension phenotypes. Permutational multivariate analysis of variance (PERMANOVA) was performed for statistical comparisons of samples in the two groups. P value was adjusted by the Benjamini and Hochberg false discovery rate (FDR). Abbreviations: IDH: isolated diastolic hypertension; ISH, isolated systolic hypertension; SDH: systolic-diastolic hypertension. [file Image_2.tif]
