# Supplementary material for: Gut mycobiome dysbiosis contributes to the development of hypertension and its response to immunoglobulin light chains
Source: Front Immunol. 2022 Dec 29;13:1089295. doi: 10.3389/fimmu.2022.1089295 (PMC9835811; doi:10.3389/fimmu.2022.1089295)
Supplement: Supplementary file 3 [file Table_1.doc]

**Table S1 Comparison of nutrient intake among groups**

| **Nutrients** | pre-HTN (n=38) | HTN  (n=46) | NT  (n=34) | *p* value |
| --- | --- | --- | --- | --- |
| Energy (kcal/d) | 2485.33±305.25 | 2347.75±357.10 | 2298.74±394.17 | 0.134 |
| Protein (g/d) | 67.99±10.99 | 65.19±8.99 | 66.98±16.58 | 0.063 |
| Fat (g/d) | 157.78±25.93 | 153.84±16.74 | 156.77±22.61 | 0.077 |
| Carbohydrate (g/d) | 128.65±28.90 | 117.35±27.36 | 138.68±44.86 | 0.607 |
| Fiber (g/d) | 6.95±1.59 | 6.79±1.17 | 6.47±1.57 | 0.737 |
| Vitamin A | 255.25±108.66 | 209.77±43.36 | 289.63±173.78 | 0.056 |
| Carotene | 11.32±4.04 | 8.97±3.94 | 10.45±3.85 | 0.107 |
| Retinol (μg/d） | 697.46±94.85 | 665.21±75.23 | 692.74±105.85 | 0.910 |
| Cholesterol (mg/d) | 633.72±199.93 | 641.34±186.74 | 655.85±222.94 | 0.894 |
| Vitamin B1 (mg/d) | 0.42±0.13 | 0.44±0.14 | 0.39±0.15 | 0.227 |
| Vitamin B2 (mg/d) | 1.12±0.33 | 1.02±0.24 | 1.04±0.34 | 0.055 |
| Vitamin C (mg/d) | 51.59±26.49 | 43.92±25.32 | 52.42±23.02 | 0.155 |
| Vitamin E (mg/d) | 49.34±5.24 | 49.14±6.34 | 48.94±5.12 | 0.114 |
| Calcium (mg/d) | 893.40±245.64 | 824.34±240.25 | 934.01±249.49 | 0.974 |
| Phosphorus (mg/d) | 853.34±191.44 | 895.92±199.01 | 861.23±243.65 | 0.634 |
| Potassium (mg/d) | 1343.62±277.32 | 1273.32±232.53 | 1269.52±296.52 | 0.785 |
| Sodium (mg/d) | 826.93±269.47 | 765.04±266.33 | 744.71±224.78 | 0.534 |
| Magnesium (mg/d) | 205.74±17.04 | 204.41±15.53 | 204.39±30.23 | 0.994 |
| Iron (mg/d) | 29.24±11.55 | 24.16±11.37 | 27.35±11.37 | 0.917 |
| Zinc (mg/d) | 17.85±5.45 | 18.35±5.65 | 19.33±4.33 | 0.093 |
| Selenium (μg/d） | 40.23±4.45 | 40.55±5.65 | 40.25±7.12 | 0.313 |
| Copper (mg/d) | 1.46±0.27 | 1.57±0.27 | 1.57±0.27 | 0.065 |
| Manganese (mg/d) | 2.45± 0.35 | 2.66 ± 0.26 | 2.68±0.26 | 0.890 |

ANOVA test was performed.

Abrreviations: HTN, hypertension; NT, normotension; pre-HTN, pre-hypertension.
