# Supplementary material for: Gut mycobiome dysbiosis contributes to the development of hypertension and its response to immunoglobulin light chains
Source: Front Immunol. 2022 Dec 29;13:1089295. doi: 10.3389/fimmu.2022.1089295 (PMC9835811; doi:10.3389/fimmu.2022.1089295)
Supplement: Supplementary file 4 [file Table_2.doc]

**Table S2 Dominated fungal species in samples**

| **Species** | **Pre-HTN (%)** | **HTN(%)** | **NT(%)** |
| --- | --- | --- | --- |
| g_Aspergillus;s_cibarius | 0.6814158 | 0.1732196 | 0 |
| g_Aspergillus;s_domesticus | 0.3407079 | 0.0866098 | 0 |
| g_Aspergillus;s_flavus | 0.1244053 | 0.0597196 | 0 |
| g_Aspergillus;s_halophilicus | 0.0000474 | 0 | 0 |
| g_Aspergillus;s_penicillioides | 0.00000677 | 0 | 0 |
| g_Aspergillus;s_sydowii | 0.00000097 | 0 | 0 |
| g_Candida;s_albicans | 0.00000019 | 0 | 0 |
| g_Candida;s_metapsilosis | 0.00000006 | 0 | 0 |
| g_Candida;s_orthopsilosis | 0.000679 | 0.000361 | 0.0000909 |
| g_Candida;s_parapsilosis | 1.6321921 | 2.8739696 | 5.9043212 |
| g_Candida;s_tropicalis | 1.0102763 | 0.4103065 | 2.2581121 |
| g_Cladosporium;s_delicatulum | 0.02409123 | 0.0243884 | 0.12018383 |
| g_Cladosporium;s_halotolerans | 0.00401521 | 0.00406473 | 0.02003064 |
| g_Cladosporium;s_sphaerospermum | 0.00080304 | 0.00081295 | 0.00400613 |
| g_Malassezia;s_arunalokei | 0.00016061 | 0.00016259 | 0.00080123 |
| g_Malassezia;s_globosa | 0.00005354 | 0.0000542 | 0.00026708 |
| g_Malassezia;s_japonica | 0.0159816 | 0.0463935 | 0.2002303 |
| g_Malassezia;s_Malassezia_obtusa | 0.00002677 | 0.0000271 | 0.00013354 |
| g_Malassezia;s_restricta | 0.00000669 | 0.00000677 | 0.00003338 |
| g_Saccharomyces;s_cerevisiae | 0.00000067 | 0.00000068 | 0.00000334 |
| g_Starmerella;s_etchellsii | 0.6814158 | 0.1732196 | 0 |
| g_Wallemia;s_sebi | 0.0722737 | 0.0731652 | 0.3605515 |

Abrreviations: HTN, hypertension; NT, normotension; pre-HTN, pre-hypertension.
