# Supplementary material for: Gut mycobiome dysbiosis contributes to the development of hypertension and its response to immunoglobulin light chains
Source: Front Immunol. 2022 Dec 29;13:1089295. doi: 10.3389/fimmu.2022.1089295 (PMC9835811; doi:10.3389/fimmu.2022.1089295)
Supplement: Supplementary file 5 [file Table_3.doc]

**Table S3 Correlations between fungal genera and white blood cells**

| **Genus** | **White blood cells** | **r-value** | **p-value** |
| --- | --- | --- | --- |
| *Mortierella* | White blood cell count | -0.001 | 0.996 |
|  | Monocytes proportion | 0.024 | 0.881 |
|  | Lymphocytes proportion | -0.034 | 0.836 |
|  | Neutrophils proportion | 0.031 | 0.850 |
|  | Eosinophiles proportion | -0.173 | 0.285 |
|  | Basophils Proportion | -0.089 | 0.583 |
| *Malassezia* | White blood cell count | 0.129 | 0.429 |
|  | Monocytes proportion | 0.118 | 0.467 |
|  | Lymphocytes proportion | 0.163 | 0.315 |
|  | Neutrophils proportion | 0.107 | 0.512 |
|  | Eosinophiles proportion | 0.235 | 0.145 |
|  | Basophils Proportion | -0.155 | 0.338 |
| f_Debaryomycetaceae, other | White blood cell count | -0.102 | 0.532 |
|  | Monocytes proportion | -0.024 | 0.885 |
|  | Lymphocytes proportion | 0.194 | 0.230 |
|  | Neutrophils proportion | -0.163 | 0.313 |
|  | Eosinophiles proportion | -0.144 | 0.376 |
|  | Basophils Proportion | -0.065 | 0.692 |

Pearson correlation analysis was performed.
