# Supplementary material for: Gut mycobiome dysbiosis contributes to the development of hypertension and its response to immunoglobulin light chains
Source: Front Immunol. 2022 Dec 29;13:1089295. doi: 10.3389/fimmu.2022.1089295 (PMC9835811; doi:10.3389/fimmu.2022.1089295)
Supplement: Supplementary file 6 [file Table_4.doc]

**Table S4 Correlations between fungal genera and hypertension associated parameters**

| **Genus** | **Hypertension associated parameters** | **r-value** | **p-value** |
| --- | --- | --- | --- |
| *Mortierella* | Age | -0.117 | 0.468 |
|  | BMI | -0.043 | 0.789 |
|  | Estimated glomerular filtration rate | 0.009 | 0.955 |
| *Malassezia* | Age | 0.135 | 0.402 |
|  | BMI | -0.052 | 0.745 |
|  | Estimated glomerular filtration rate | -0.116 | 0.475 |
| f_Debaryomycetaceae, other | Age | -0.160 | 0.317 |
|  | BMI | -0.052 | 0.745 |
|  | Estimated glomerular filtration rate | -0.081 | 0.621 |

Pearson correlation analysis was performed.
